# Prevalence of cardiovascular risk factors, the use of statins and of aspirin in Takayasu Arteritis

**DOI:** 10.1038/s41598-021-93416-0

**Published:** 2021-07-13

**Authors:** Charlotte Laurent, Sergio Prieto-González, Pierre Belnou, Fabrice Carrat, Olivier Fain, Azeddine Dellal, Maria C. Cid, José Hernández-Rodríguez, Arsène Mekinian

**Affiliations:** 1 AP-HP, Service de Médecine Interne and Inflammation-Immunopathology-Biotherapy Department (DMU i3), Hôpital Saint-Antoine, Sorbonne Université, 75012 Paris, France; 2 Vasculitis Research Unit, Department of Autoimmune Diseases, Hospital Clínic of Barcelona, Institut D’Investigacions Biomèdiques August Pi i Sunyer (IDIBAPS), University of Barcelona, Barcelona, Spain; 3 INSERM, Institut Pierre Louis d’épidémiologie et de Santé Publique (IPLESP UMRS 1136), Sorbonne Universités, UPMC Université Paris 06, 75012 Paris, France; 4 Unité de Santé Publique, Hôpital Saint Antoine, Assistance Publique-Hôpitaux de Paris, 75012 Paris, France; 5 Service de Rhumatologie, Hôpital Montfermeil, Montfermeil, France

**Keywords:** Diseases, Medical research

## Abstract

The aim of this study was to assess the prevalence of cardiovascular risk factors in TAK, to describe the use of aspirin and statins and the risk factors associated with vascular ischemic complications and relapses. We conducted a retrospective study on TAK patients diagnosed between 2010 and 2018. Demographic, clinical, laboratory data and treatments were evaluated at diagnosis and during the follow-up. We included fifty-two TAK patients with median age 37.5 years [range 16–53] and 43 (83%) women. At diagnosis, cardiovascular risk factors were present in 32 (62%) patients: hypertension (n = 20, 38%), hyperlipidemia (n = 8, 15%), tobacco use (n = 16, 31%). During the median 4-year follow-up [range 0.1–17 years], 17 (33%) patients had at least one ischemic event and 15 (29%) patients needed endovascular procedure. Whereas TAK patients with cardiovascular risk factors were more frequently on statins and anti-hypertensive drugs, they have higher rates of cumulative ischemic complications (5 (24%) versus 21 (67%); *p* = 0.004), but similar rates of aspirin-treated patients. Patients who have developed vascular ischemic events were more frequently smokers (53% versus 20%; *p* = 0.03). The vascular complication-free survival was not significantly different in TAK patients with or without statins or aspirin at diagnosis. During the follow-up, 27 (52%) patients had at least one relapse, and the relapse-free survival was not significantly different in patients treated with statins or aspirin. Cardiovascular risk factors in TAK have to be strictly controlled since these risk factors could be associated with increased risk of ischemic complications.

## Introduction

Takayasu arteritis (TAK) is a large-vessel vasculitis of unknown origin characterized by vascular granulomatous inflammation. TAK affects young females during the second or third decades of life and predominantly involves the aorta and its branches, leading to wall thickening, stenosis and vessel occlusion^1^. Vascular manifestations are related to stenotic or occlusive arterial lesions, which can affect the aortic arch, descending thoracic or abdominal aorta, supra-aortic trunks, renal, abdominal, coronary and pulmonary arteries. TAK has been associated with significantly increased morbidity and mortality, and the identification of patients with poor prognosis could improve their management^2,3^. A recent French study of 318 patients identified three risk factors associated with death and vascular ischemic complications: (1) progressive disease course at diagnosis; (2) thoracic aorta involvement; and (3) hypertensive retinopathy^4^. Various studies have shown that cardiovascular disease is the most common cause of death in patients with TAK, with cardiomyopathy and ischemic stroke among the most frequent complications causing mortality. However, the benefit of treating cardiovascular risk factors have been poorly evaluated^2,3,5–8^. Aspirin was shown to be associated with a decrease of cardiovascular ischemic events during the follow-up and more recently, statin use has been associated with a decrease in relapse rate^9,10^.

The aim of this study was to assess the prevalence of cardiovascular risk factors in TAK, to describe the use of aspirin and statins and determine risk factors associated with vascular ischemic complications and relapses.

## Patients and methods

### Patients

We conducted a retrospective bicentric study from two university centers (Saint Antoine Hospital, France and Barcelona University center, Spain) between January 2010 and January 2018. All consecutive patients with TAK followed in both university centers (ACR and/or Ishikawa criteria modified by Sharma), with at least 12 months of follow-up, were included in the study^11–13^. Eight patients were over 50 years old at TAK vasculitis diagnosis, but had no sign of temporal artery vasculitis and four of them had a negative temporal artery biopsy; presence of signs suggestive of TAK vasculitis above 50 years; presence of some characteristics features of TAK such as renal artery and subclavian artery stenosis in all patients.

Patients' characteristics were recorded at baseline and during the follow-up (at 3, 6, 12, 24, 36 months and last visit) as follows: gender, associated diseases, age, constitutional and vascular symptoms, cardiovascular risk factors and therapies (Anti-platelet agents, statins, antihypertensive drugs, antivitamin K) and immunosuppressive therapies. Cardiovascular risk factors were considered for each patient at diagnosis and at the different times of the follow-up: arterial hypertension, obesity, diabetes mellitus, tobacco use. Arterial hypertension is defined as systolic blood pressure greater than or equal to 140 mmHg and /or diastolic blood pressure greater than or equal to 90 mmHg. Obesity is defined as abnormal or excessive fat accumulation that presents a risk to health. A body mass index (BMI) over 30 is obese. Diabetes mellitus is defined as a fasting plasma glucose level above 1.27 g/l two times. Laboratory data were recorded as follows: C-reactive protein (CRP), creatinine, blood counts and hepatic enzymes. Disease activity was defined using the NIH scale and relapse was defined as NIH ≥ 2 in patients with remission since at least 2 months^1^. Vascular complications were defined as either clinical ischemic event (ischemic stroke, heart infarction, limb ischemia defined by extremity pain, limb vascular claudication or gangrene) and/or the need of vascular procedures at any time during the follow-up.

Informed consent was obtained from all subjects and from a parent and/or legal guardian from patients under 18. This retrospective study was approved by the Research Ethics Committee (Comité des personnes, Hôpital Saint-Antoine, Ile-de-France V) and the study was performed in accordance with the Helsinki Declaration.

### Statistical analysis

Data are expressed as median (ranges) for quantitative variables, and number (%) for categorical variables. Continuous data were compared by Mann Whitney or Kruskal–Wallis test for more than two groups and categorical data by Fisher’s exact test as appropriate considering the value distribution. Event-free survival was defined as the time from the date of TA diagnosis and the date of first vascular complication, death (of any cause), or last follow-up. Survivals were estimated using the Kaplan–Meier method and compared using log-rank test. Factors associated with the time-to-event outcomes (vascular ischemic complications and relapses) were evaluated using Cox models, with estimation of hazards ratios and their 95% confidence interval. All tests were two-tailed and *p* < 0.05 was considered statistically significant. Statistical analyses involved using GraphPad Prism v7.00 for Windows (GraphPad Software, La Jolla, CA, USA) and R software.

## Results

### Patients’ characteristics

We identified fifty-two TAK patients with a median age at diagnosis 37.5 [16–53] years including43 (83%) women.. Patients’ ethnicity included Caucasian (n = 26), sub-Sahara African or Afro-Caribbean (n = 15), Asian (n = 5) and others ethnicities (n = 6). At TAK diagnosis, 47 (90%) patients had vascular symptoms, 35 (67%) patients had constitutional symptoms with increased acute-phase reactants in 37 (71%) patients (Table 1). The NIH scale at diagnosis was 3 [1–4]. During the follow-up, 44 (85%) patients received glucocorticoids, 38 (73%) patients received additional immunosuppressive drugs [including methotrexate (n = 28), azathioprine (n = 11), cyclophosphamide (n = 3), mycophenolate mofetil (n = 1)] and 37 (67%) patients were treated with biological-targeted therapies, which include TNF-α blockers [infliximab (n = 11), etanercept (n = 1), adalimumab (n = 1)], tocilizumab (n = 35) and rituximab (n = 3).

**Table 1:** Comparison of Takayasu arteritis (TAK) patients with and without ischemic events during the follow-up.

	All TAK patients (n = 52)	Patients with ischemic events (n = 17)	Patients without ischemic events (n = 35)
**Characteristics at diagnosis**			
Age (years) (medians; ranges)	37.5 [16–63]	41 [22–63]	36 [16–62]
Women (n; %)	43 (83)	12 (71)	31 (89)
Vascular signs	41 (79)	13 (76)	28 (80)
Fever / Weight loss (n; %)	30 (58)	9 (53)	21 (60)
C-reactive protein (mg/L) (median; range)	43 [0–230]	53 [1–230]	38 [1–200]
NIH disease activity score at diagnosis (median, range)	3 [1–4]	3 [1–4]	3 [1–4]
**Cardiovascular risk factors at diagnosis**			
Diabetes mellitus (n; %)	1 (2)	0	1 (2.9)
Hypertension (n; %)	19 (37)	8 (47)	11 (31)
Smokers (n; %)	16 (31)	9 (53)	7 (20)*
Hyperlipidemia (n; %)	8 (15)	5 (29)	3 (9)
Body mass index	25.8 [17.6–38]	20.9 [17.6–29.4]	27.1 [17.6–37.9]
**TAK treatments**			
Glucocorticoids (n, %) at diagnosis	40 (82)	12/16 (75)	28/33 (85)
Additional immunosuppressants (n, %) at any time	28 (62)	11/12 (92)	17/33 (52)*
Biological-targeted therapies (n, %) at any time	35 (67)	10/15 (67)	25/35 (71)
**Cardiovascular risk factors therapies**			
Aspirin at diagnosis (n; %)	33 (65)	15 (88)	18 (53)*
Aspirin at any time (n; %)	36 (69)	15 (88)	21 (62)
Anticoagulants	5 (9.6)	3 (18)	2 (6)
Statins at diagnosis (n; %)	13 (27)	8 (47)	5 (16)*
Statins at any time (n; %)	16 (32)	8 (47)	8 (24)
Antihypertensive drugs at any time (n, %)	22 (42)	12 (71)	10 (31)*
ACE inhibitor at any time (n, %)	16 (33)	9 (53)	7 (22)*
β-blockers at any time (n, %)	11 (22)	5 (29)	6 (19)
**Outcome**			
Relapses	27 (52)	9 (53)	18 (51)
Follow-up (years)	4.2	4.1	4.3

Values are medians with ranges and numbers with frequencies.

*p < 0.05.

### Prevalence of cardiovascular risk factors

At diagnosis, cardiovascular risk factors were present in 32 (62%) patients: arterial hypertension (n = 20, 38%), hyperlipidemia (n = 8, 15%), and 16 (31%) patients were smokers. At diagnosis of TAK, cardiovascular risk factors therapies (previously introduced) consisted of antihypertensive drugs (n = 22; 42%) with angiotensin-converting enzyme inhibitors (n = 17; 33%), beta-blockers (n = 11; 21%), statins (n = 13; 29%), aspirin (n = 36; 69%) and anticoagulants (n = 3; 6%). Aspirin was given at a median dose of 75 mg/day and statins included atorvastatin (n = 12, at a median dose of 20 mg/day), simvastatin (n = 2, at 10 mg/day) and rosuvastatin (n = 1, at 10 mg/day).

Comparing TAK patients without anycardiovascular risk factor at diagnosis (n = 21) to those with at least one risk (n = 31), there was no difference in NIH scale and median C-reactive protein levels; therelapse rates during the follow-up were not significantly different between TAK with and without any cardiovascular risk factors. TAK patients with at least one cardiovascular risk factor tended to be more of male sexes (1 (5%) versus 8 (26%); *p* = 0.08), and have higher IMC (28 kg/m^2^ [18–39] versus 20 [17–29]; *p* = 0.008). Whereas TAK patients with at least one cardiovascular risk factors were more frequently on statins and anti-hypertensive drugs, with similar rates of aspirin-treated patients (13 (62%) versus 19 (61%); *p* = 0.2), the cumulative rates of ischemic complications were higher in those with cardiovascular risk factors (5 (24%) versus 21 (67%); *p* = 0.004),.

### Factors associated with ischemic events

**Figure 1:**
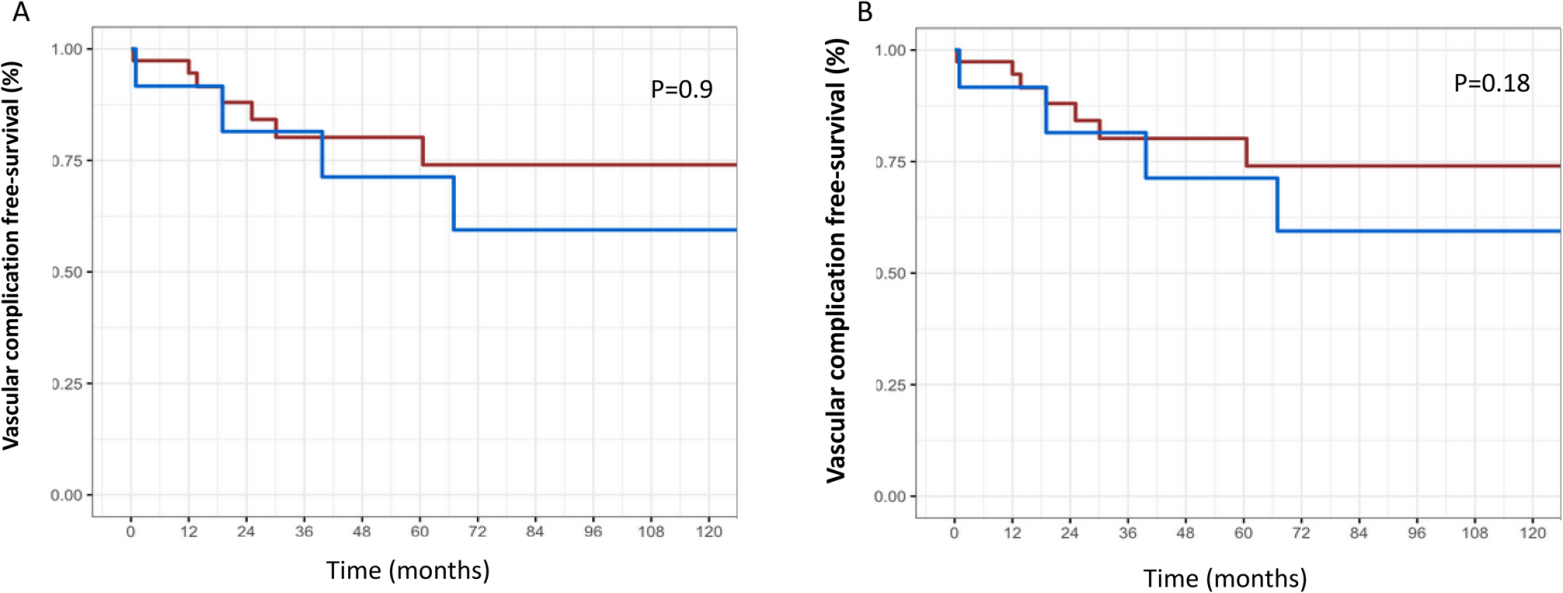
Vascular complication free-survival with (red) and without (blue) statins.

During the median 4-year follow-up [range 0.1–17 years], 17 (33%) patients developed at least one vascular ischemic event, and among them, 15 (29%) patients needed endovascular procedures. TAK patients who developed at least one ischemic event were more frequently smokers (53% versus 20%; *p* = 0.03), tended to have more often hyperlipidemia (29% versus 9%; *p* = 0.09), and lower body mass index (20.9 versus 27.1; *p* = 0.08). The number of patients treated with glucocorticoids did not differ between TAK with and without ischemic events during the follow-up. However, the number of patients on glucocorticoids combined to an additional immunosuppressant drug experiencing an ischemic event was higher (92% versus 52%, *p* = 0.02) (Table 1). Patients who developed an ischemic event were more frequently taking antihypertensive drugs (71% versus 31%, *p* = 0.008), aspirin (88% versus 53%, *p* = 0.02) and statins (47% versus 16%, *p* = 0.02) than those without ischemic events (Table 1). Nevertheless, the vascular complication-free survival was not significantly different in TAK without and those taking statins (log rank = 0.9) (Fig. 1A,B). The vascular complication-free survival without and under low-dose aspirin at diagnosis was not significantly different (log rank = 0.18) (Fig. 1A,B), with hazard ratio for vascular ischemic complications under aspirin at 2.8 [0.61;12.8] (*p* = 0.18).

### Factors associated with relapse

**Figure 2:**
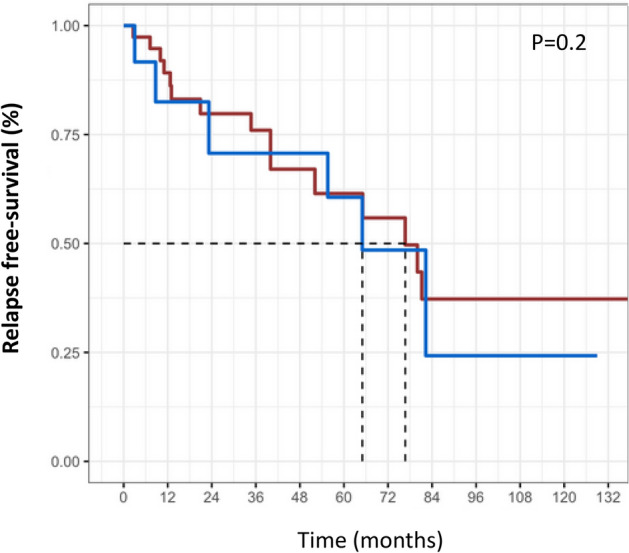
Relapse free-survival with (red) and without (blue) statins.

During a median 4-year follow-up [range 0.1–17 years], 27 (52%) patients had at least one relapse, and 26 (96%) of them needed a drug change. Comparing patients with and without relapse during the follow-up, CRP levels tended to be higher in patients with relapses (50 mg/l versus 20 mg/l, *p* = 0.06), whereas there was not any other difference at baseline of TAK features and cardiovascular risk factors frequencies. Relapsing patients were more frequently on glucocorticoids at diagnosis (88% versus 64%, *p* = 0.049) and during the follow-up (96% versus 72%, *p* = 0.02), and received more often a biological-targeted therapy (89% versus 44%, *p* = 0.0009) (Table 2). The relapse-free survival was not significantly different in TAK patients with and without statins with hazard ratio at 1.13 [0.43; 2.9] (*p* value = 0.8 and log rank = 0.2) (Fig. 2).

**Table 2:** Comparison of Takayasu arteritis (TAK) patients with and without relapse during the follow-up.

	All TAK patients (n = 52)	Patients with relapses (n = 27)	Patients without relapses (n = 25)
**Characteristics at diagnosis**			
Age (years) (medians; ranges)	37.5 [16–63]	42 [18–53]	35 [16–50]
Females (n; %)	43 (83%)	21 (77%)	22 (88%)
Vascular signs	41 (79)	20 (74)	21 (84)
Fever / Weight loss (n; %)	30 (58)	15 (55)	15 (60)
C-reactive protein (mg/L) (medians; ranges)	43 [0–230]	50 [1–230]	20 [1–144]
NIH disease activity score at diagnosis (medians, ranges)	3 [1–4]	3 [1–4]	3 [1–4]
**Cardiovascular risk factors at diagnosis**			
Diabetes mellitus (n; %)	1 (2)	0	1 (4)
Hypertension (n; %)	19 (37)	10 (37)	9 (38)
Smokers (n; %)	16 (31)	3 (11)	13 (52)*
Hyperlipidemia (n; %)	8 (15)	5 (19)	3 (12)
Body mass index	25.8 [17.6–38]	25.8 [19–32]	25.8 [17.6–38]
**TAK treatments**			
Glucocorticoids at diagnosis	40 (82)	24 (88)	16 (64)*
Glucocorticoids at any time	44 (85)	26 (96)	18 (72)*
Additional immunosuppressants (n, %) at any time	28 (62)	21 (78)	7 (28)*
Biological-targeted therapies at any time (n, %)	35 (67)	24 (89)	11 (44)*
**Cardiovascular risk factors therapies**			
Aspirin (n; %)	36 (69)	19 (70)	17 (68)
Anticoagulants	5 (9.6)	3 (11)	2 (8)
Statins (n; %)	16 (32)	8 (32)	8 (32)
Antihypertensive drugs (n, %)	25 (50)	16 (59)	9 (36)
ACE inhibitor (n, %)	15 (29)	10 (37)	5 (20)
Betablockers (n, %)	17 (33)	12 (44)	5 (20)
Follow-up (years)	4.2	4.3	4.0

Values are medians with ranges and numbers with frequencies.

*p < 0.05.

## Discussion

Two of the major objectives of the treatment of TAK include preventing relapses and ischemic events due to disease activity or progression of TAK vascular lesions. Previous studies have shown that a persistent active disease and progressive course are major factors of vascular disease progression and ischemic complications. While usual cardiovascular risk factors are also clearly involved in the progression of vascular lesions, and intricate with the TAK-related arterial progression, the management of these risk factors is not yet well established.

One of the major findings of our study is that TAK patients with increased cardiovascular risk factors have increased risk of vascular ischemic events despite relatively young age (median 37.5 years old in this study). Our findings confirm the previous data about the increased atherosclerosis in this vasculitis, and enforce the implication of common cardiovascular factors. Our data are consistent with a recent study in which the prevalence of cardiovascular factors was similar in this young subset of patients^8^. In this previous study, tobacco use was associated with a lower vascular ischemic events-free survival, but the impact of other cardiovascular risk factors was not demonstrated^8^. The mechanism of vascular ischemic events in TAK can thus be explained by active TAK vascular disease, but probably also in relation with increased rates of atherosclerosis from commune origin.

The development of ischemic lesions in TAK could be secondary to the vasculitis activity or fibrotic progression from atherosclerosis lesions, and the exact origin of the ischemic lesions could be challenging to determine. Benefits of the management of the cardiovascular risk factors, as the use of anti-platelet agents and statins, in preventing new vascular stenotic or occlusive lesions could be especially interesting to determine, independently from the use of immunosuppressive strategies. Several cardiovascular risk factors are more frequent among TAK patients who experience ischemic events as shown in our cohort of TAK patients, especially hypercholesterolemia and tobacco use. In consequence, these patients were more frequently treated with statins and aspirin. Although disease activity and the occurrence of relapses were not significantly different between patients treated for cardiovascular risk factors and those untreated, the increased use of glucocorticoids and additional immunosuppressive agents in patients experiencing more relapses and in those developing ischemic complications could be explained by the fact that these relapsing patients exhibit an increased disease activity from the baseline, which led physicians to treat them more aggressively.

A previous study showed a protective role of aspirin for the prevention of ischemic events in TAK patients, and patients with ischemic complications used significantly less anti-platelet agents (14.3%) than those without ischemic lesions (82.4%)^9^. In our study the aspirin use was not significantly associated with better vascular complication-free survival, However drawing a definitive conclusion is difficult due to its retrospective design and the relatively small number of patients on aspirin. The recently updated EULAR recommendations for the management of large-vessel vasculitis recommends the use of anti-platelet agents in TAK after individual evaluation, based on the degree of vessel stenosis and other cardiovascular risk factors, as usually considered in general population^14^.

The occurrence of relapses is a challenge in TAK, as more than 40% of patients experience at least one relapse during their follow-up^2^. Few risk factors for relapse in TAK have been described, and our group recently reported that male sex, increased C-reactive protein levels and the presence of carotidynia were associated with increased rates of relapse ^8^. In the present study, higher acute-phase reactants at diagnosis, reflecting the intense systemic inflammatory response, seem to be associated with an increased risk of relapses and higher glucocorticoid requirements in TAK patients^15^. The increased use of glucocorticoids was also accompanied by a more frequent requirement of biological-targeted therapies in TAK patients.

Statins can inhibit T cell responses by suppressing Th-1 responses and pro-inflammatory cytokines production^16^. The Th-1 and Th-17 cytokines drive inflammation in TAK, and the increase of Th-1 and Th-17 cells activation correlates with disease activity^17^. In a previous study of 74 TAK patients, the statins use was associated with a lower risk of relapse (71% versus 36%, *p* = 0.003)^10^. Despite the potential immunomodulatory effects of statins, in our study the use of statins was not associated with a better relapse–free survival andsimilar negative results were observed in a cohort of 54 patients with biopsy-proven GCA^18^.

Our study has several limitations, in particular the retrospective design and relatively low number of ischemic events in our limited study population. The usual management of cardiovascular risk factors has still to be strictly warranted since it is well-known that, similarly to individuals with other autoimmune diseases, TAK patients have a higher risk of developing an accelerated atherosclerotic disease than the general population^19^. One of our study’s most important limitations is that we could not discriminate vascular ischemic events related to uncontrolled disease activity from those due to atherosclerotic lesions, whereas the statins and anti-platelet agents use could differently affect the prognosis in those different situations.

## Conclusion

TAK patients, despite their young age, have an increased prevalence of common cardiovascular risk factors which could participate to increased rates of atherosclerosis in this vasculitis. These various cardiovascular risk factors have been associated with increased rates of ischemic complications. Cardiovascular risk factors in TAK have to be strictly controlled since they could be associated with increased risk of ischemic complications.
